# Multicentric small cell neuroendocrine neoplasm of the renal pelvis and ureter with concomitant focal high-grade urothelial carcinoma of the ureter: A case report

**DOI:** 10.4103/0970-1591.44273

**Published:** 2008

**Authors:** John S. Banerji, Anila Korula, Jayalakshmi B. Panicker

**Affiliations:** Department of Urology, Christian Medical College, Vellore-632 004, Tamil Nadu, India; 1 Pathology, Christian Medical College, Vellore-632 004, Tamil Nadu, India

**Keywords:** Multicentric, neuroendocrine tumor, renal pelvis, urothelial tumor

## Abstract

Malignant small cell neuroendocrine tumors of the pelvi-calyceal system are rare, and even more uncommon is their occurrence with concomitant transitional cell carcinoma, in the same renal unit. We present such a case for its unique presentation.

## INTRODUCTION

Although most small cell carcinomas arise in the tracheobronchial tree, they are increasingly being reported in extra-pulmonary sites like the esophagus, cervix, parotid and in the genitourinary tract like the prostate and bladder.

Capella *et al*.,[1] reported the first case of small cell tumor of the renal pelvis. There have been no documented reports of simultaneously occurring malignant neuroendocrine tumor of the renal pelvis with transitional cell carcinoma (TCC).

## CASE REPORT

A 55-year-old gentleman had right loin pain for six months. He had no lower urinary tract symptoms (LUTS), fever or hematuria. On evaluation he was found to have microscopic hematuria and positive urine cytology. An ultrasound revealed right hydroureteronephrosis and a middle calyceal lesion with perinephric stranding.

**Figure 1:**
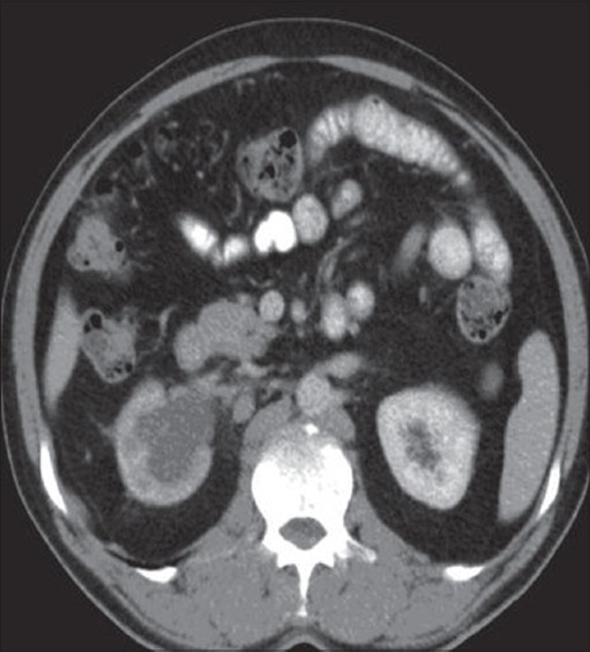
CT showing right hydronephrosis with a small pelvic lesion

A computed tomography (CT) scan revealed a small 15×15 mm pelvi-calyceal lesion, along with a mid-ureteric thickening [Figure 1] and several large para-aortic and interaortocaval nodes (size 5×4×3.5 cm), along with a small thrombus in the renal vein, with extension to IVC. However, histology did not mention any tumor thrombus.

A working diagnosis of TCC was made and he underwent a right retrograde pyelogram, a ureterorenoscopy followed by right open nephroureterectomy with a bladder cuff.

Retrograde pyelogram (RGP) showed a complete cutoff of contrast at the mid-ureter with inability of glide wire to be negotiated beyond it [Figure 7]. Ureterorenoscopy (URS) revealed a papillary tumor at the mid-ureter.

Intraoperatively the mid-ureter was densely adherent to the retroperitoneum and there were large interaortocaval and pre-caval nodes, which were also removed.

### Histopathology

**Figure 2:**
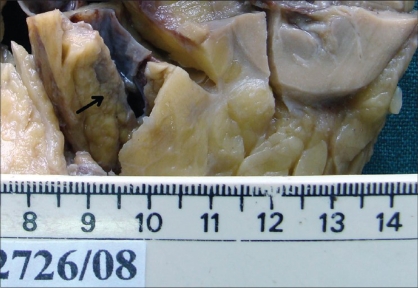
Tumor in the renal pelvis

**Figure 3:**
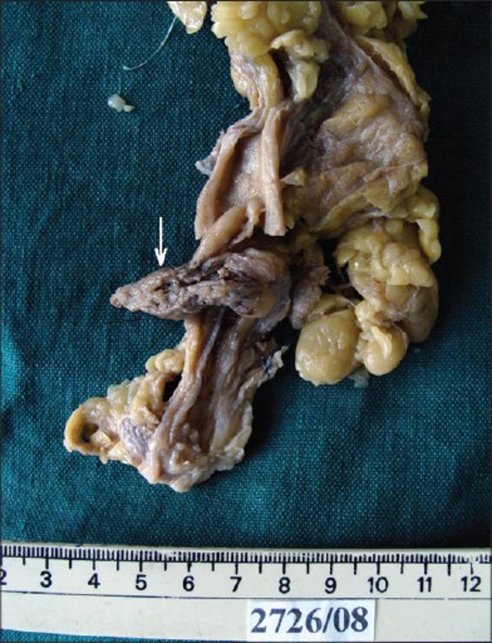
Tumor in the ureter

On gross examination, the kidney showed a well-defined tumour in the interpolar region [Figure 2] measuring 1×1×2 cm with a firm grey white cut surface and a separate tiny nodule, 0.5 cm, 0.2 cm away from the tumor. The adjacent renal parenchyma appeared unremarkable. The ureter showed a tumor [Figure 3] measuring 7×1×1.5 cm filling the lumen, 12 cm away from the renal hilum with a similar cut surface. There were areas of necrosis and hemorrhage.

**Figure 4:**
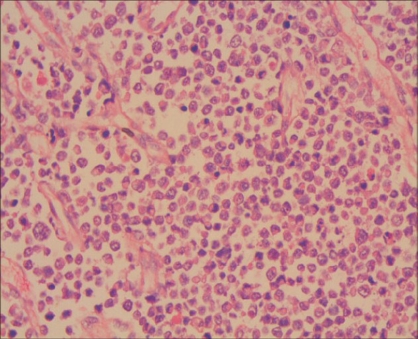
Small cell neuroendocrine tumor in the renal pelvis (H&E, x400)

Renal pelvis was infiltrated by the tumor which was composed of dispersed single cells and sheets of small to medium-sized cells with round, hyperchromatic mitotically active nuclei and scant eosinophilic cytoplasm [Figure 4]. There were binucleate forms with apoptosis. The tumor cells were separated by fibrocollagenous tissue septa. The tumor was infiltrating the perihilar adipose tissue and renal medulla.

**Figure 5:**
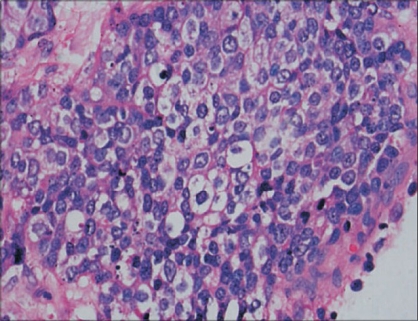
High-grade transitional cell carcinoma in the ureter (H&E, ×400)

The wall of the ureter was infiltrated by tumor as described above with areas of necrosis. There were nests of polygonal cells with vesicular nuclei, prominent nucleoli and clear cytoplasm [Figure 5]. Hilar veins had no histological evidence of tumor thrombus.

**Figure 6:**
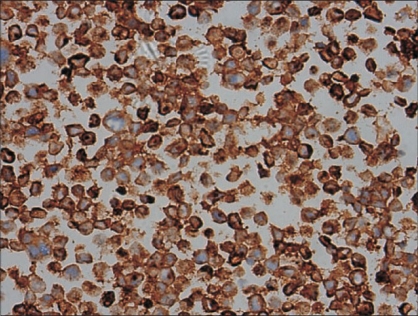
Small cell neuroendocrine carcinoma - Tumor cells positive for Synaptophysin (x400)

Separately sent interaortocaval lymph nodes showed reactive hyperplasia and no tumor. Immunohistochemical markers showed cytoplasmic positivity for neuron specific enolase (NSE) and synaptophysin [Figure 6] in the small cell component and the neoplastic cells were negative for cytokeratin.

**Figure 7:**
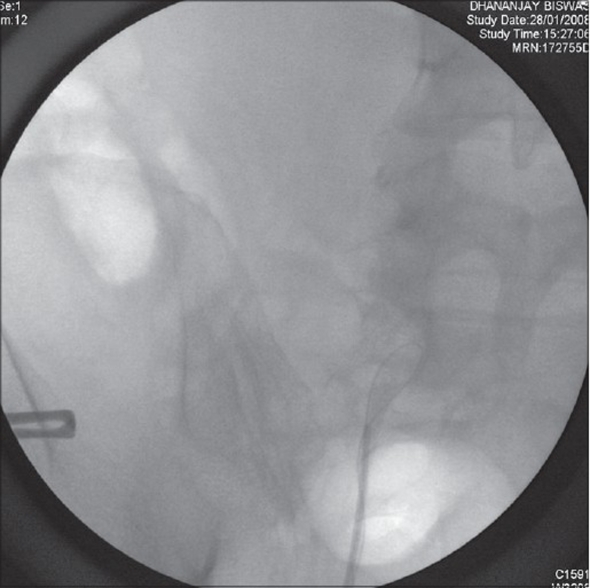
RGP Image

## DISCUSSION

Primary malignant small cell neuroendocrine tumors comprise a group of highly malignant tumors, which hitherto are difficult to characterize, both clinically and histologically.

Neuroendocrine tumors in the urinary tract have been described in the literature and they range from carcinoid tumors to small cell carcinomas. These tumors are more common in the renal pelvis, even though there are reports of neuroendocrine tumors occurring in the renal parenchyma, ureter and urinary bladder.[2] The histogenesis of these tumors remains controversial and warrants further studies. One view is that they are of urothelial origin with neuroendocrine differentiation and the other view is that they originate from the neuroendocrine cells present in the renal pelvis. Some of the authors suggest that these tumors originate from the entrapped neural crest in the kidney during embryogenesis.[3] These tumors arise from undifferentiated stem cells with multipotential differentiation towards urothelial or squamous cell lineage and when these elements are present, they tend to be of high grade.[4,5] This tumor type is characterized by an aggressive clinical course with early metastasis. The overall survival rate for patients with small cell carcinoma of the bladder with local disease has been reported as low as 8%.[6] The usual sites of metastasis are lymph nodes and bone. The differential diagnoses include malignant lymphoma, lymphoepithelioma like carcinoma, plasmacytoid carcinoma, poorly differentiated urothelial carcinoma and primitive neuroectodermal tumor, from which this tumor can be differentiated by immunohistochenical markers.[7]

This particular patient had a tumor in the renal pelvis with a small satellite tumor adjacent to it and another in the ureter, both showing histological and immunohistochemical features of neuroendocrine origin. In addition, the tumor in the ureter contained focal nests of high-grade urothelial carcinoma. To our knowledge, this is the first case in the literature of multicentric high-grade neuroendocrine tumor in the urothelial tract with concomitant high-grade urothelial carcinoma of the ureter.

It is well known that the kidney can be the site of tumors with endocrine-paracrine differentiation. Tumors of the diffuse endocrine system collectively known as carcinoids, have been reported.[8,9] It is, however, much more important to rule out a primary lung tumor metastasizing to the kidney. Chest X-ray and CT thorax were normal thus ruling out primary pulmonary malignancy.

The key to the diagnosis of the small cell component is ultrastructure demonstration of secretory neuroendocrine granules and immunohistochemistry for synaptophysin and neuron-specifice enolase.

Due to the rarity of genitourinary small cell neuroendocrine neoplasms, there are no well-defined protocols for adjuvant therapy. The discussion at the multidisciplinary meeting was that gemcitabine and carboplatin would be offered for six cycles for this patient. There have been studies proving the use of carboplatinum, etoposide and vincristine for small cell carcinoma of the lung.[10] It is reasonable to expect improved survivals with platinum-based chemotherapy in combination with irinotecan,[11] as there is histological similarity to small cell carcinoma of the lung.
